# Supplementary material for: MUON: multimodal omics analysis framework
Source: Genome Biol. 2022 Feb 1;23:42. doi: 10.1186/s13059-021-02577-8 (PMC8805324; doi:10.1186/s13059-021-02577-8)
Supplement: Supplementary file 1 — Additional file 1. Figure S1 [file 13059_2021_2577_MOESM1_ESM.pdf]

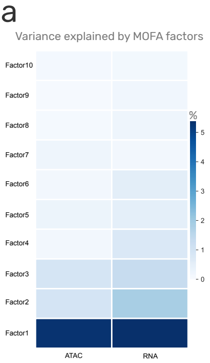

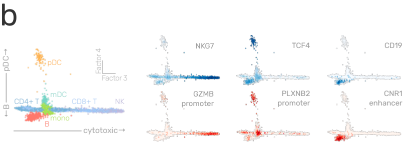

Fig. S1 MOFA factors estimates and variance explained.
a Variance explained by each of 10 MOFA factors in each modality of simultaneous scRNA-seq and scATAC-seq profiling of PBMCs. Colour denotes the percentage of variance explained.
b MOFA factors estimated from simultaneous scRNA-seq and scATAC-seq profiling of PBMCs, with cells coloured by either left: coarse-grained cell type; or right: gene expression (in blue) and peak accessibility (in red). Displayed genes and peaks are selected to represent cell type-specific variability along the Factor3 – Factor4 axis.
