# Supplementary material for: MUON: multimodal omics analysis framework
Source: Genome Biol. 2022 Feb 1;23:42. doi: 10.1186/s13059-021-02577-8 (PMC8805324; doi:10.1186/s13059-021-02577-8)
Supplement: Supplementary file 2 — Additional file 2. Review history [file 13059_2021_2577_MOESM2_ESM.docx]

Review History

**First round of review**

**Reviewer 1**

**Were you able to assess all statistics in the manuscript, including the appropriateness of statistical tests used?**

There are no statistics in the manuscript.

**Were you able to directly test the software?**

Yes

**Comments to author:**

SUMMARY

This article describes MUON, a software package for analysing multiomics data. MUON is built around MuData, a new data structure for holding multiomics data. This data structure is designed to be interoperable and compatible with multiple languages including R and Julia, although Python is the primary platform. A MuData object contains multiple arbitrary layers where each layer is a modality. In the Python implementation layers are self-contained AnnData objects, a commonly used format for scRNA-seq data with an existing community. This structure allows hierarchical storage of data where some fields (such as mean expression) are part of an individual layer while other (such as cell annotations or join embeddings) are relevant to the whole object. As well as the MuData object, MUON includes functionality for analysing the multiomics data it contains, particularly joint analysis such as MOFA. This package addresses a clear need in the community and has the potential to improve workflows for analysts and developers.

MAJOR CONCERNS

I think the MuData object has the potential to be important infrastructure for the community to build around. My main criticism of the paper (rather than the tool itself) is the lack of technical detail around how MuData is implemented. This makes it difficult to assess some of the claims in the paper and would be important information for potential readers looking to use and build on MuData. Some of the things it would be great to read more about are:

- How do you manage slicing, particularly for features which are different across modalities?

- Does the object/package know about specific types of modalities (RNA, ATAC etc.), or is every layer equivalent?

- How do you avoid loading the whole dataset into memory (is it disk-backed or something else)?

- How are objects converted between languages? Are the objects identical or is some information lost? If it is not consistent it would be great to see a mapping showing what is/isn't transferred in either direction.

A lot of this technical detail already exists in the documentation but as MuData is the primary innovation here I think it would be fitting and useful for it to be included in the paper as well.

MINOR CONCERNS

- Please clarify the wording around the non-Python implementations. You state that "MUON comes with dedicated libraries to create, read and write MuData objects from Python, R and Julia". This is not quite right as there is no implementation of an R MuData object (I didn't check the Julia package). What you can do with the R package is interface with .h5mu files and get Seurat/MultiAssayExperiment objects, which is probably more useful currently but not quite what the text implies.

- It might be helpful to clarify if MUON is more for single-cell multiomics or multiomics in general. The abstract/introduction is fairly general but some of the other sections imply a single-cell focus.

- MOFA plays a big role in the analysis and the description of the capabilities of MUON. It would be helpful to have a one-sentence description of MOFA somewhere in the paper for readers who are unfamiliar with the method.

- The comparison table states that Seurat and MultiAssayExperiment object are unable to work without loading all data into memory but there are disk-backed versions of both of these objects. How is this different to what MuData/AnnData do? Possibly I am misunderstanding what this refers to, if so please clarify.

- As the table compares objects, maybe replacing the muon and scanpy headings with MuData and AnnData would be a better fit?

- It's not quite clear what the roman numerals in the Figure 2B caption refer to, it would be helpful to have these numerals in the figure so the link is clearer. It would also be helpful to have a description for the matrices labelled W and Z in the caption.

- Figure 3A shows the same information twice on different MOFA dimensions. Is there a reason to show both sets of plots or would just one (or maybe the MOFA UMAP) be enough? This would also save some space in the figure which only just fits on the page currently.

- I completely missed the tutorial links the first time I looked at the paper and documentation (I assumed they were part of the package documentation but they are a separate website). These are a great resource so I encourage you to mention them specifically in the paper and link to them in other places (package README, documentation etc.).

- Please make the code for the analysis available in a public repository. I know most of it is available in the tutorials but those are likely to be updated over time and it would be good to have a record of exactly what was done here.

TYPOS AND GRAMMAR

- I find words ("and") easier to read than symbols ("&", "+") (but ask the editor about the journal style guide)

- There is at least one reference to a "MUON container" which I think should be MuData?

- There are a few sentences that might be missing words, for example "using existing toolchains that build {on} this data standard" (P3L27)

SOFTWARE

- Software code is publicly available ✅

- Code is covered by a clear software license ✅

- I was able to install the Python and R packages ✅

- I was able to run the examples for the Python package ✅

- I was not able to run the examples for the R package ❌

- The ReadH5MU function shown in the R README is not exported by the package so isn't available to the user. Please make sure all functionality is exported and documented.

- Please add a link to the documentation site to the GitHub README so it's easy for users to find. I thought there was no documentation until I saw the link in the paper.

- Some of the output in the documentation didn't match the output I got from running the code or sometimes what was described in the text (see the "Variable mappings" section). Please check that the documentation is consistent and up to date.

- I also noticed that some of the example vignettes just had comments instead of showing the output. It would be great if there was an example dataset and output for these so users can check it works.

SUGGESTIONS/QUESTIONS

NOTE: These are not comments on the paper, just ideas I had/things I was curious about. Feel free to not reply to these if you don't think it's worth the time.

- Would it be possible to avoid the update command for MuData objects? I can see how this is necessary but I can see a lot of potential pain for users who forget to do an update.

- Have you considered moving MuData to a separate package like AnnData is separate from Scanpy? Probably some effort now but I think in the long term it would be easier for other developers.

- Is there any way to track links between features in different modalities? For example it might be useful to be able to subset by gene on the whole object and get back RNA expression, any associated ATAC peaks and protein expression (or whatever is available).

As I mentioned in the summary I think MuData could be important infrastructure for the community. There are a few improvements I would like to see for the paper but I hope they are not too much work and you are able to get this published soon.

**Reviewer 2**

**Were you able to assess all statistics in the manuscript, including the appropriateness of statistical tests used?**

There are no statistics in the manuscript.

**Were you able to directly test the software?**

Yes

**Comments to author:**

This manuscript presents the muon data structure for on-disk representation of multimodal omics data based on the HD5 file specification. It is intended as a cross-language data standard for out-of-memory storage and analysis of multimodal data, with initial support provided for Python, Julia, and R. The cross-language aspect is potentially useful and impactful, however the current implementation is preliminary and it remains unclear when it will reach the stage of maturity required for broad adoption. I think the manuscript and its table incorrectly tries to position muon as an alternative to Seurat and MultiAssayExperiment, when its actual implementation is, appropriately, as a plugin to the R data structures defined by those packages that can provide more cross-platform support. In summary I support the effort to provide a cross-platform data standard for multiomic data, but significant work on the software remains to achieve this in a way that will be broadly adopted, and more thorough review of the software it is meant to work alongside is needed. This review focuses on the R component.

Major:

1. The statement "MuData files can be seamlessly accessed from different programming languages, including Python [16], R [17], and Julia [18]." isn't currently correct for R because although the package contains a function ReadH5MU, it is not exported and therefore not easily available or known to the end user. The R package `muon.R` version 0.0.0.9000 exports a single function (WriteH5MU).

2. The package attempts to support existing widely-used Bioconductor data structures SummarizedExperiment, SingleCellExperiment, and MultiAssayExperiment. This is a good approach because these data structures already have a large user base and ecosystem of analysis packages around them, so if Muon could interface these with other programming languages it would be a useful addition. But this is a difficult goal and I'm not convinced that it is achieved yet. For example:

3. The WriteH5MU function supports two multiomics data classes: Seurat and MultiAssayExperiment. The MultiAssayExperiment support is hard-coded to support only SummarizedExperiment and SingleCellExperiment, and would require new development to support any other of the range of classes that are compatible with MultiAssayExperiment. A more object-oriented approach that supports any data class inheriting from SummarizedExperiment, or any data class meeting certain requirements, would be a major re-design but would also greatly extend the utility of the R component.

4. Some checks and informative error messages are needed to identify unsupported data classes. For example:

> coad <- curatedTCGAData::curatedTCGAData("COAD", "*", version = "2.0.1", dry.run = FALSE)

> WriteH5MU(coad, "coad_test.mu")

Error in dataset_create_pl$set_chunk(chunk_dims) : HDF5-API Errors:

error #000: ../../../src/H5Pdcpl.c in H5Pset_chunk(): line 2025: all chunk dimensions must be positive

class: HDF5

major: Invalid arguments to routine

minor: Out of range

5. After using WriteH5MU and muon:::ReadH5MU for MultiAssayExperiment, some of the sampleMap information (the edge list which maps assay profiles to samples in colData) is lost, which may lead to overall data loss. In this example the values in the colnames column were changed during the operation, e.g., TCGA-OR-A5J1 vs TCGA-OR-A5J1-01A-11R-A29S-07 (original):

library(muon)

library(MultiAssayExperiment)

data(miniACC)

WriteH5MU(miniACC, "test.mu")

test <- muon:::ReadH5MU("test.mu", "MultiAssayExperiment")

sampleMap(test)

sampleMap(miniACC)

6. In some variables, NAs are converted to character "NA" data rather than kept coded as missing values `NA` within R.

table(colData(test)$pathologic_stage, useNA = "always")

\# VS

table(colData(miniACC)$pathologic_stage, useNA = "always")

7. The software does not seem to support genomic coordinate-based data, which should be discussed and acknowledged as a shortcoming.

8. The table and text on limitations of Seurat and MultiAssayExperiment are unaware of available functionality - I have not checked the same for scanpy but it may be as well. Specifically:

The statement that Seurat and MultiAssayExperiment "require loading the full dataset into the working memory, which precludes dealing with larger datasets, unless HDF5-based extensions are used to store the data [15,16]." and this summary in the table is incorrect or at least misleading. These packages, like muon, depend on external packages for representation of H5 files, but otherwise support HDF5 solutions to on-disk representation and analysis without loading full datasets into memory (Seurat with its H5Seurat file format, MultiAssayExperiment with its default support of DelayedArray objects as assay components and its saveHDF5MultiAssayExperiment and loadHDF5MultiAssayExperiment functions.

The table is incorrect in that serialization of MultiAssayExperiment is implemented in H5 (saveHDF5MultiAssayExperiment) and CSV (exportClass) formats out of the box, in addition to RDS.

The table is incorrect in that MultiAssayExperiment does support importing data from python-based (H5AD) formats via the Bioconductor ecosystem (HDF5Array::H5ADMatrix). It is worth noting that MultiAssayExperiment supports any assay data structures that meet a minimum of functionality, so it supports most other data structures in the Bioconductor ecosystem and inherits this functionality from HDF5Array automatically without re-implementing it.

9. Coding details

These are issues that would prevent the package from passing checks for entry into CRAN or Bioconductor, but are not currently being checked because the package is only on GitHub. For publication in a top journal, all the software packages should be submitted to recognized repositories that subject the code to continuous integration.

Although MultiAssayExperiment is used within package code (ie, setMethod("WriteH5MU", "MultiAssayExperiment", …) MultiAssayExperiment is listed in the Suggests field rather than Imports field of the NAMESPACE.

The software uses library calls within the code, but should use `importFrom` or `::` to define dependencies.

The functions don't provide any user-facing documentation (there is no man/ directory).

The code could be made more interpretable and maintainable with helper functions that reduce the amount of repetition

**Authors Response**

**Point-by-point responses to the reviewers’ comments:**

Reviewer #1

This article describes MUON, a software package for analysing multiomics data. MUON is built around MuData, a new data structure for holding multiomics data. This data structure is designed to be interoperable and compatible with multiple languages including R and Julia, although Python is the primary platform. A MuData object contains multiple arbitrary layers where each layer is a modality. In the Python implementation layers are self-contained AnnData objects, a commonly used format for scRNA-seq data with an existing community. This structure allows hierarchical storage of data where some fields (such as mean expression) are part of an individual layer while other (such as cell annotations or join embeddings) are relevant to the whole object. As well as the MuData object, MUON includes functionality for analysing the multiomics data it contains, particularly joint analysis such as MOFA. This package addresses a clear need in the community and has the potential to improve workflows for analysts and developers.

*Thank you for your comments and suggestions and this careful review of our manuscript and the associated software.*

MAJOR CONCERNS

I think the MuData object has the potential to be important infrastructure for the community to build around. My main criticism of the paper (rather than the tool itself) is the lack of technical detail around how MuData is implemented. This makes it difficult to assess some of the claims in the paper and would be important information for potential readers looking to use and build on MuData. Some of the things it would be great to read more about are:

- How do you manage slicing, particularly for features which are different across modalities?

- Does the object/package know about specific types of modalities (RNA, ATAC etc.), or is every layer equivalent?

- How do you avoid loading the whole dataset into memory (is it disk-backed or something else)?

- How are objects converted between languages? Are the objects identical or is some information lost? If it is not consistent it would be great to see a mapping showing what is/isn't transferred in either direction.

A lot of this technical detail already exists in the documentation but as MuData is the primary innovation here I think it would be fitting and useful for it to be included in the paper as well.

*We acknowledge the omission of technical details in the manuscript itself. In response to this comment, we have added a section Implementation of MuData in Methods (page 6), in which major design considerations and implementation details on the serialisation are provided. Briefly:*

*Slicing MuData objects along the samples dimension subsets the requested samples in each modality as well as in the multimodal annotations. A feature set of a multimodal object is essentially a concatenation of features of individual modalities, and slicing along the features dimension allows to get a MuData object which is a subset of the multimodal dataset with requested features in their respective modalities.*

*MuData objects as well as individual modalities (AnnData objects) allow for storing unstructured data. This can be used for recording modality- or method-specific information (e.g. genome assembly version or variance explained by principal components) and in turn can be used by analysis tools such as muon.*

*The MuData object structure itself just encompasses AnnData objects as individual modalities, which do not possess any modality-defining traits. This also signifies that MuData is a generic solution for the multimodal omics data infrastructure.*

*To enable memory efficiency, disk backing is implemented for MuData objects in the same way it works for AnnData objects. This means that count matrices in individual modalities are not loaded into memory but are fetched from disk when needed.*

*MuData objects are serialized by default to HDF5-based .h5mu files, with individual modalities stored inside in a way compatible (in fact, fully compliant) with .h5ad serialisation. Libraries to operate with HDF5 files exist for many programming languages making this file format accessible from other programming environments.*

*We also provide libraries for Julia and R programming languages to deserialize .h5mu files. In the case of Julia, we implemented native AnnData and MuData objects. For R, we deserialize data into objects widely used for omics analysis such as MultiAssayExperiment and Seurat. As outlined in the section Comparison of MuData with alternative data formats, MuData allows to store more information than the current versions of these R data types so some information such as multimodal embeddings might not be deserialized automatically (but of course can be accessed, e.g. manually, from the file). In the revised versions of the R packages (see also responses to Reviewer 2) we have also added warnings if some data can’t be deserialized automatically. We also note that native access to .h5mu files from the R environment can be achieved with HDF5 libraries (rhdf5, hdf5r) as well as using R-Python bridge via reticulate as, for instance, described here: https://theislab.github.io/scanpy-in-R/.*

*We acknowledge that since .h5ad and .h5mu are not the native formats for R frameworks such as Seurat, currently there's no standard how to serialize auxiliary information stored in the R object — and, conversely, deserialize this information back from the .h5ad/.h5mu file (this information can be stored in various places of .h5ad/.h5mu files). R libraries that we implemented to work with .h5ad and .h5mu files — MuDataMAE and MuDataSeurat — can become scaffolds for such standards. This is now discussed in the Implementation of MuData section (page 6).*

MINOR CONCERNS

- Please clarify the wording around the non-Python implementations. You state that "MUON comes with dedicated libraries to create, read and write MuData objects from Python, R and Julia". This is not quite right as there is no implementation of an R MuData object (I didn't check the Julia package). What you can do with the R package is interface with .h5mu files and get Seurat/MultiAssayExperiment objects, which is probably more useful currently but not quite what the text implies.

*Thank you, we have clarified in the text that this refers to MuData files (.h5mu).*

*The I/O operations with .h5mu files in R are now implemented in two separate libraries — MuData and MuDataSeurat — that were also briefly described above as well as mentioned below. We also denoted that these R libraries de-serialize .h5mu files to MultiAssayExperiment/Seurat objects in the table in the section Comparison of MuData with alternative data formats of the Methods.*

- It might be helpful to clarify if MUON is more for single-cell multiomics or multiomics in general. The abstract/introduction is fairly general but some of the other sections imply a single-cell focus.

*MuData is a general solution for multimodal data with MUON as associated multimodal omics analysis framework. Nevertheless, we agree that the focus of the manuscript is on single-cell multi-omics applications, which we feel is one of the most important use cases and which we describe in the Application of MUON to single-cell multi-omics data section. We revised the text to clarify it (e.g. page 2, paragraph 3). Please note that the examples and notebooks also include non-single-cell use cases (e.g. chronic lymphocytic leukemia data analysis, https://muon-tutorials.readthedocs.io/en/latest/CLL.html).*

- MOFA plays a big role in the analysis and the description of the capabilities of MUON. It would be helpful to have a one-sentence description of MOFA somewhere in the paper for readers who are unfamiliar with the method.

*In response to this comment, we have included a brief description in the results section (page 4, paragraph 4). We also refer the reader to more detailed reviews on multi-omics integration methods in the Discussion.*

- The comparison table states that Seurat and MultiAssayExperiment object are unable to work without loading all data into memory but there are disk-backed versions of both of these objects. How is this different to what MuData/AnnData do? Possibly I am misunderstanding what this refers to, if so please clarify.

*Thank you for pointing this out. While we agree that there are disk-backed versions for Seurat and MultiAssayExperiment, these existing implementations have major limitations. Firstly, we now denote that disk backing in R depends on additional libraries such as SeuratDisk or HDF5Array.*

*Secondly, while we deem a discussion of R libraries not to be the focus of this manuscript, here we would like to argue that disk backing for Seurat and MAE is currently a second-class citizen. For both Seurat+SeuratDisk and MultiAssayExperiment+HDF5Array disk backing is only possible through HDF5 files — and is not suported for RDS files, the default R serialization format.*

*That means that Seurat objects serialized to RDS files, as per tutorials, cannot take advantage of disk backing. Moreover, SeuratDisk is currently still an experimental package that hasn’t seen any contributions for the last half a year (the last commit is 163f1aa at the time of writing) and that states in its documentation for the loading function LoadH5Seurat() that by default, it loads the entire object into memory, meaning that the user still has to choose assays (modalities) to be loaded into memory and then actively opt out of loading individual slots. A Seurat object is then created with chosen modalities and slots in memory, i.e. while SeuratDisk does provide convenience of creating files from parts of the data in the HDF5 file, there is no data backed on disk in these objects. More so, since Seurat strictly defines the dgCMatrix type to be used for sparse counts, currently it’s impossible to implement a third-party library to enable disk backing for matrices in Seurat objects.*

*Regarding MultiAssayExperiment+HDF5Array, backing a matrix is enabled by storing an external HDF5 file in addition to an RDS file with the rest of the data from the MultiAssayExperiment object. Multiple files are hence to be kept and shared “together”. While we cannot consider this a fair serialization strategy, it does allow users to work with MultiAssayExperiment objects which have count matrices stored on disk, and this is now reflected in the corresponding section of the table. Note that MuData allows users to work with a single .h5mu file, and to use disk backing for the count matrices of individual modalities.*

- As the table compares objects, maybe replacing the muon and scanpy headings with MuData and AnnData would be a better fit?

*Thank you for your suggestion. We now refer to the data formats in the header of the table to make it clear we compare data objects rather than analytical functionality.*

- It's not quite clear what the roman numerals in the Figure 2B caption refer to, it would be helpful to have these numerals in the figure so the link is clearer. It would also be helpful to have a description for the matrices labelled W and Z in the caption.

*We added these numerals to the figure as well as we denoted that W and Z refer to weights and factors matrices in the figure caption.*

- Figure 3A shows the same information twice on different MOFA dimensions. Is there a reason to show both sets of plots or would just one (or maybe the MOFA UMAP) be enough? This would also save some space in the figure which only just fits on the page currently.

*Thank you for this suggestion, we have moved the dimensionality reduction plot with factors 3 and 4 to supplementary figures.*

- I completely missed the tutorial links the first time I looked at the paper and documentation (I assumed they were part of the package documentation but they are a separate website). These are a great resource so I encourage you to mention them specifically in the paper and link to them in other places (package README, documentation etc.).

*Thank you for the kind remarks on the MUON tutorials and for noting that they should be linked in more places. We have added a respective link in the Methods section as well as in the MUON documentation and in the MUON repository. Here we would also note that we have created a webpage with these links aggregated and tutorials prominently referenced — https://gtca.github.io/muon — but in the manuscript, we prefer to directly refer to the primary sources.*

- Please make the code for the analysis available in a public repository. I know most of it is available in the tutorials but those are likely to be updated over time and it would be good to have a record of exactly what was done here.

*The MUON tutorials on Multiome and CITE-seq data demonstrate a superset of analysis results presented in the manuscript. Together with this submission we also provide the snapshot of the code for mudata and muon as well as for the tutorials: https://doi.org/10.5281/zenodo.5557543.*

TYPOS AND GRAMMAR

- I find words ("and") easier to read than symbols ("&", "+") (but ask the editor about the journal style guide)

- There is at least one reference to a "MUON container" which I think should be MuData?

- There are a few sentences that might be missing words, for example "using existing toolchains that build {on} this data standard" (P3L27)

*Thank you for pointing out the typos and excessive vocabulary (“MUON container”), which might be confusing. We corrected the updated manuscript accordingly and carefully revised spelling and grammar.*

*Regarding the symbol use, Chromium Single Cell Multiome ATAC + Gene Expression is a full name for the solution for combined single-cell RNA-seq and ATAC-seq by 10X Genomics (https://www.10xgenomics.com/products/single-cell-multiome-atac-plus-gene-expression), and it only seems appropriate to refer to it by its name even though it does contain a plus symbol. We also use the ampersand in the context of “scRNA & scATAC” when referring to Multiome and Multiome-like solutions for combined gene expression and chromatin accessibility profiling as it is typically used in titles and shorthand expressions to indicate this refers to a single item.*

SOFTWARE

- Software code is publicly available ✅

- Code is covered by a clear software license ✅

- I was able to install the Python and R packages ✅

- I was able to run the examples for the Python package ✅

- I was not able to run the examples for the R package ❌

- The ReadH5MU function shown in the R README is not exported by the package so isn't available to the user. Please make sure all functionality is exported and documented.

*As noted above, we have now superseded the muon.r R package and we now provide two libraries — MuData and MuDataSeurat. Each of these packages now correctly exports two reading functions — ReadH5MU and ReadH5AD — as well as two writing functions — WriteH5MU and WriteH5AD. These changes will improve the robustness of the R interface; see also responses to Reviewer 2.*

- Please add a link to the documentation site to the GitHub README so it's easy for users to find. I thought there was no documentation until I saw the link in the paper.

*Thank you, in addition to the documentation link that has been hidden behind the “docs” shield in the GitHub README.md file, we also now link documentation explicitly.*

- Some of the output in the documentation didn't match the output I got from running the code or sometimes what was described in the text (see the "Variable mappings" section). Please check that the documentation is consistent and up to date.

*Thank you. We have updated the Variable mappings section in the documentation to match the current versions of muon and mudata.*

- I also noticed that some of the example vignettes just had comments instead of showing the output. It would be great if there was an example dataset and output for these so users can check it works.

*We’ve made muon tutorials available at https://muon-tutorials.readthedocs.io/. They are rendered Jupyter notebooks, which exemplify analysis steps and provide textual and graphical output on the same page, and they are also available in the GitHub repository. We now link them in the muon documentation and in the Methods section as well. We also note since the source of these notebooks is open, they can be imported into and explored in web-based environments such as Google Colab. We have also added vignettes as part of the documentation for the new R packages (MuDataMAE, MuDataSeurat), and these vignettes have been executed and compiled to provide output that users can check with.*

SUGGESTIONS/QUESTIONS

NOTE: These are not comments on the paper, just ideas I had/things I was curious about. Feel free to not reply to these if you don't think it's worth the time.

- Would it be possible to avoid the update command for MuData objects? I can see how this is necessary but I can see a lot of potential pain for users who forget to do an update.

*This is definitely doable if we just focus on adding/removing AnnData objects as modalities but there is no way of detecting when AnnData objects are changed in-place (e.g. obs_names and var_names attributes). We might consider consistency checks and automatic updates for MuData objects but doing a full update every time might be too slow, and we currently do not have any fast and robust way to do a consistency check.*

- Have you considered moving MuData to a separate package like AnnData is separate from Scanpy? Probably some effort now but I think in the long term it would be easier for other developers.

*As outlined above, this has been done, and mudata is now implemented as a separate Python library. We hope that this change will make it even easier for developers to build new tools on top of MuData.*

- Is there any way to track links between features in different modalities? For example it might be useful to be able to subset by gene on the whole object and get back RNA expression, any associated ATAC peaks and protein expression (or whatever is available).

*Links between individual features in different modalities can be stored in the .varp slot of MuData objects. We anticipate this is going to be used for new functionalities in muon and other packages. Peak annotation, which contains peak→gene links, from the Multiome output can also be stored as a Pandas data frame in the .uns slot, as it is demonstrated in the tutorials.*

As I mentioned in the summary I think MuData could be important infrastructure for the community. There are a few improvements I would like to see for the paper but I hope they are not too much work and you are able to get this published soon.

*Thank you for your kind words and for your elaborate comments that have helped us to make improvements not only to the manuscript but also to the software itself. We hope MuData and muon will help to enable multimodal omics analysis at scale.*

Reviewer #2

This manuscript presents the muon data structure for on-disk representation of multimodal omics data based on the HD5 file specification. It is intended as a cross-language data standard for out-of-memory storage and analysis of multimodal data, with initial support provided for Python, Julia, and R. The cross-language aspect is potentially useful and impactful, however the current implementation is preliminary and it remains unclear when it will reach the stage of maturity required for broad adoption. I think the manuscript and its table incorrectly tries to position muon as an alternative to Seurat and MultiAssayExperiment, when its actual implementation is, appropriately, as a plugin to the R data structures defined by those packages that can provide more cross-platform support. In summary I support the effort to provide a cross-platform data standard for multiomic data, but significant work on the software remains to achieve this in a way that will be broadly adopted, and more thorough review of the software it is meant to work alongside is needed. This review focuses on the R component.

*Thank you for your comments and your time to read the manuscript. Before addressing the individual points raised below, we would like to point out that MuData is an HDF5-based data standard for multimodal data, which facilitates cross-language access. Muon itself is a Python framework for multimodal omics data analysis. As the reference MuData data standard implementation is also based on Python, we acknowledge that user workflows when working with .h5mu files might be slightly different when using other programming languages. While we did our best to provide .h5mu I/O support in the linked R libraries, we acknowledge that not all features are exposed to R and Julia. These limitations are now discussed in the section Implementation of MuData.*

*In response to this review, the muon.r library is now superseded by 2 R packages: MuData with MultiAssayExperiment support and MuDataSeurat with Seurat support. We hope these packages address the outlined shortcomings of muon.r, and we include the point-by-point response below.*

Major:

1. The statement "MuData files can be seamlessly accessed from different programming languages, including Python [16], R [17], and Julia [18]." isn't currently correct for R because although the package contains a function ReadH5MU, it is not exported and therefore not easily available or known to the end user. The R package `muon.R` version 0.0.0.9000 exports a single function (WriteH5MU).

*We thank the reviewer for highlighting this issue. The ReadH5MU function is now correctly exported.*

*We would also like to note here that since HDF5 libraries are available for many programming languages, MuData files can in principle be accessed from any of them. For R specifically, there exist at least two implementations of such libraries including rhdf5, available in Bioconductor, and hdf5r, available in CRAN*.

2. The package attempts to support existing widely-used Bioconductor data structures SummarizedExperiment, SingleCellExperiment, and MultiAssayExperiment. This is a good approach because these data structures already have a large user base and ecosystem of analysis packages around them, so if Muon could interface these with other programming languages it would be a useful addition. But this is a difficult goal and I'm not convinced that it is achieved yet. For example:

3. The WriteH5MU function supports two multiomics data classes: Seurat and MultiAssayExperiment. The MultiAssayExperiment support is hard-coded to support only SummarizedExperiment and SingleCellExperiment, and would require new development to support any other of the range of classes that are compatible with MultiAssayExperiment. A more object-oriented approach that supports any data class inheriting from SummarizedExperiment, or any data class meeting certain requirements, would be a major re-design but would also greatly extend the utility of the R component.

*We have redesigned our implementation to dispatch on the type of the object. All classes inheriting from SummarizedExperiment can now be exported to an H5MU file. However, in the most basic case where a class-specific implementation is not available, only features supported by SummarizedExperiment will be exported, namely the assays, colData, and rowData.*

4. Some checks and informative error messages are needed to identify unsupported data classes. For example:

> coad <- curatedTCGAData::curatedTCGAData("COAD", "*", version = "2.0.1", dry.run = FALSE)

> WriteH5MU(coad, "coad_test.mu")

Error in dataset_create_pl$set_chunk(chunk_dims) : HDF5-API Errors:

error #000: ../../../src/H5Pdcpl.c in H5Pset_chunk(): line 2025: all chunk dimensions must be positive

class: HDF5

major: Invalid arguments to routine

minor: Out of range

*We attempted to provide better error handling. We note that in this particular instance, the coad object contains several RaggedExperiment objects, which are not supported by muon (see also our response to point 7).*

5. After using WriteH5MU and muon:::ReadH5MU for MultiAssayExperiment, some of the sampleMap information (the edge list which maps assay profiles to samples in colData) is lost, which may lead to overall data loss. In this example the values in the colnames column were changed during the operation, e.g., TCGA-OR-A5J1 vs TCGA-OR-A5J1-01A-11R-A29S-07 (original):

library(muon)

library(MultiAssayExperiment)

data(miniACC)

WriteH5MU(miniACC, "test.mu")

test <- muon:::ReadH5MU("test.mu", "MultiAssayExperiment")

sampleMap(test)

sampleMap(miniACC)

*We now write and restore the sample mappings.*

6. In some variables, NAs are converted to character "NA" data rather than kept coded as missing values `NA` within R.

table(colData(test)$pathologic_stage, useNA = "always")

\# VS

table(colData(miniACC)$pathologic_stage, useNA = "always")

*NA values are not supported by HDF5. While it is possible to build custom support for NA-containing arrays by using an additional masking array, support for NAs in the Python Pandas library is experimental and serialization/deserialization of NA-containing arrays is not supported by the Python AnnData package. Since muon primarily builds on AnnData, we cannot implement cross-language support for NA values. The R package is primarily intended to facilitate exchange of data between the languages and implementing a solution that only works in R would not serve this goal. For a purely R-based workflow, an R-specific serialization format like RDS or RDA might be more suitable. We also note that count data with high levels of sparsity is typically represented as sparse arrays, which are handled properly across languages.*

*We now include a note in the documentation of WriteH5MU that NAs are not supported.*

7. The software does not seem to support genomic coordinate-based data, which should be discussed and acknowledged as a shortcoming.

*That is correct. The Python AnnData package, which muon builds upon, has no explicit support for coordinate-based annotation, although one could imagine an ad-hoc solution using specially crafted strings in the .var DataFrame (the AnnData equivalent to colData). Since our focus is on the Python ecosystem and the R package is intended to facilitate data exchange between the languages, we can only support the intersection of features of the respective platforms.*

*We now emit a warning if an unsupported format is passed to WriteH5MU.*

8. The table and text on limitations of Seurat and MultiAssayExperiment are unaware of available functionality - I have not checked the same for scanpy but it may be as well. Specifically:

The statement that Seurat and MultiAssayExperiment "require loading the full dataset into the working memory, which precludes dealing with larger datasets, unless HDF5-based extensions are used to store the data [15,16]." and this summary in the table is incorrect or at least misleading. These packages, like muon, depend on external packages for representation of H5 files, but otherwise support HDF5 solutions to on-disk representation and analysis without loading full datasets into memory (Seurat with its H5Seurat file format, MultiAssayExperiment with its default support of DelayedArray objects as assay components and its saveHDF5MultiAssayExperiment and loadHDF5MultiAssayExperiment functions.

The table is incorrect in that serialization of MultiAssayExperiment is implemented in H5 (saveHDF5MultiAssayExperiment) and CSV (exportClass) formats out of the box, in addition to RDS.

The table is incorrect in that MultiAssayExperiment does support importing data from python-based (H5AD) formats via the Bioconductor ecosystem (HDF5Array::H5ADMatrix). It is worth noting that MultiAssayExperiment supports any assay data structures that meet a minimum of functionality, so it supports most other data structures in the Bioconductor ecosystem and inherits this functionality from HDF5Array automatically without re-implementing it.

*We now implement disk-backed objects using HDF5Array. We note that HDF5Array uses the rhdf5 Bioconductor package for HDF5 support, whereas we previously used hdf5r from CRAN. To ensure smooth interoperability between HDF5Array and the rest of our code, we now also use rhdf5. However, rhdf5 does not yet support all HDF5 features that AnnData makes use of. We have implemented the required functionality and submitted a pull request to rhdf5 and are in touch with the rhdf5 maintainer (Mike Smith, EMBL) to get it approved. In the meantime, our fork of rhdf5 (https://github.com/ilia-kats/rhdf5/tree/attribute-references) must be used with MuData.*

*We have also updated the table with new headings, as well as changed and clarified some of its entries: we now denote in the table that additional libraries such as SeuratDisk or HDF5Array are required for this to work in R.*

*Here, we also note that technically, MultiAssayExperiment does not depend on HDF5Array but rather suggests it, so the HDF5-based serialization for matrices does require installing an additional library, in contrast e.g. to AnnData that does depend on h5py.*

*While we acknowledge that the R libraries provided by Muon currently do not provide the same functionality as their python equivalents, we would like to argue that disk backing for Seurat and MAE is a second-class citizen. For both Seurat+SeuratDisk and MultiAssayExperiment+HDF5Array disk backing is only possible through HDF5 files — and not with RDS files, a native R serialization format.*

*That means that Seurat objects serialized to RDS files, as per tutorials, cannot take advantage of disk backing. Moreover, SeuratDisk is currently still an experimental package that hasn’t seen any contributions for the last half a year (the last commit is 163f1aa at the time of writing) and that states in its documentation for the loading function LoadH5Seurat() that by default, it loads the entire object into memory, meaning that the user still has to choose assays (modalities) to be loaded into memory and then actively opt out of loading individual slots. A Seurat object is then created with chosen modalities and slots in memory, i.e. while SeuratDisk does provide convenience of creating files from parts of the data in the HDF5 file, there is no data backed on disk in these objects. More so, since Seurat strictly defines the dgCMatrix type to be used for sparse counts, currently it’s impossible to implement a third-party library to enable disk backing for matrices in Seurat objects.*

*For MultiAssayExperiment+HDF5Array, backing a matrix is enabled by storing an external HDF5 file in addition to an RDS file with the rest of the data from the MultiAssayExperiment object. Multiple files are hence to be kept and shared “together”. While we cannot consider this a fair serialization strategy, it does allow users to work with MultiAssayExperiment objects which have count matrices stored on disk, and this is now reflected in the corresponding section of the table. Note that MuData allows users to work with a single .h5mu file, and to use disk backing for the count matrices of individual modalities.*

9. Coding details

These are issues that would prevent the package from passing checks for entry into CRAN or Bioconductor, but are not currently being checked because the package is only on GitHub. For publication in a top journal, all the software packages should be submitted to recognized repositories that subject the code to continuous integration.

Although MultiAssayExperiment is used within package code (ie, setMethod("WriteH5MU", "MultiAssayExperiment", …) MultiAssayExperiment is listed in the Suggests field rather than Imports field of the NAMESPACE.

The software uses library calls within the code, but should use `importFrom` or `::` to define dependencies.

The functions don't provide any user-facing documentation (there is no man/ directory).

The code could be made more interpretable and maintainable with helper functions that reduce the amount of repetition

*We have split the muon.r package into MuData for MultiAssayExperiment support and MuDataSeurat for Seurat support. All functions are properly exported, imported, and documented. MuData will be submitted to Bioconductor upon our rhdf5 pull request approval (see response to point 8 above). We also note that R package checks finish with no errors, which can be replicated with:*

*# for MuData:*

*git clone https://github.com/pmbio/MuDataMAE*

*cd MuDataMAE*

*R CMD check .*

*# Bioconductor checks*

*R CMD BUILD . --no-build-vignettes*

*R -e "BiocCheck::BiocCheck('MuData_0.99.0.tar.gz')"*

*# for MuDataSeurat:*

*git clone https://github.com/pmbio/MuDataSeurat*

*cd MuDataSeurat*

*R CMD check .*

**Second round of review**

**Reviewer 1**

Thank you for your responses and changes to address my comments. I am happy with what you have done and am excited to see the paper published. (I slightly disagree with your assertion that HDF5 is a first class file format in Python but not R, but I am refraining from commenting on it because I don't think it affects whether the paper should be published :-))

**Reviewer 2**

Some improvements have been made, including separating MuData and MuDataSeurat, and maintaining the sampleMap when writing a MultiAssayExperiment. But I remain concerned about usability of the R software, and the commitment to submitting it to Bioconductor and CRAN, and to providing the level of documentation and ongoing development that would be required to build and maintain a user base. The pull request apparently referenced in the response (https://github.com/grimbough/rhdf5/pull/95) was merged into rhdf5 on Sept 7, but in the couple of months since, MuData has still not been submitted to Bioconductor and MuDataSeurat still has not been submitted to CRAN. With no Continuous Integration in use, I had to download and check the packages locally to determine that these packages still contain a number of issues making them difficult to use or assess. Using R 4.1.2 and Bioconductor 3.14 (full devtools::session_info() at the end).

1. 1. Checking MuData

\# for MuData:

git clone https://github.com/pmbio/MuDataMAE

cd MuDataMAE

R CMD check .

* checking for file ‘./DESCRIPTION’ ... ERROR

Required fields missing or empty:

‘Author’ ‘Maintainer’

$ R CMD BUILD . --no-build-vignettes ## note erroneously capitalized BUILD in response to reviewers /usr/lib/R/bin/Rcmd: 64: exec: BUILD: not found

$ R CMD build . --no-build-vignettes ##corrected BUILD -> build

* checking for file ‘./DESCRIPTION’ ... OK

* preparing ‘MuData’:

* checking DESCRIPTION meta-information ... OK

* checking for LF line-endings in source and make files and shell scripts

* checking for empty or unneeded directories

Omitted ‘LazyData’ from DESCRIPTION

* building ‘MuData_0.99.0.tar.gz’

If R CMD build is run with vignettes, the vignette does not build, ending with the following error, meaning the code can’t just be run by a user:

E> Quitting from lines 116-118 (Getting-Started.Rmd)

E> Error: processing vignette 'Getting-Started.Rmd' failed with diagnostics:

E> failed to open HDF5 file '/tmp/RtmpDZRrLM/Rbuild54226f0af12c/MuData/vignettes/miniacc.h5mu' E> --- failed re-building ‘Getting-Started.Rmd’

There are some significant BiocCheck notes (leaving out the less significant notes):

[1] "'LazyData:' in the 'DESCRIPTION' should be set to false or removed"

[2] "Use accessors; don't access S4 class slots via '@' in examples/vignettes."

[3] "Avoid '<<-' if possible (found 1 times)"

[4] "Avoid 'suppressWarnings'/'*Messages' if possible (found 1 times)"

2. MuDataSeurat The MuDataSeurat vignette uses two packages (SeuratData, BiocStyle) that are undeclared in the DESCRIPTION Suggests field, causing errors on a fresh containerized check. SeuratData is not in CRAN, so it requires defining remotes or googling to find where to install the package from. After resolving this, R CMD check on MuDataSeurat still has 6 warnings and 6 notes that would have to be resolved for it to be accepted into CRAN. There is an unusual “Warning in file.copy(pkgdir, vd2, recursive = TRUE) : too deep nesting”, along with more routine ones about undocumented code objects and calls to unimported functions not defined in the global environment.

3. coad example

The coad example from my previous review now gives some informative warnings about skipping RaggedExperiment objects, but it still gives a cryptic error in the end “Error in assign(mname, def, where)”:

> WriteH5MU(coad, "coad_test.mu")

Error in assign(mname, def, where) :

cannot add bindings to a locked environment

In addition: Warning messages:

1:	In .local(object, file, overwrite, ...) :

Objects of class RaggedExperiment are currently unsupported, skipping...

2:	In .local(object, file, overwrite, ...) :

Objects of class RaggedExperiment are currently unsupported, skipping...

3:	In .local(object, file, overwrite, ...) :

Objects of class RaggedExperiment are currently unsupported, skipping...

4:	In .local(object, file, overwrite, ...) :

Objects of class RaggedExperiment are currently unsupported, skipping…

This error also occurs with the object after removing the RaggedExperiment objects, suggested the authors didn’t check whether the example would now run after dropping RaggedExperiment objects.

4. discussion about genomic coordinate-based data “The software does not seem to support genomic coordinate-based data, which should be discussed and acknowledged as a shortcoming.” – this is now discussed in the response to reviewers, but still not in the manuscript. As a note, supported data classes (e.g. RangedSummarizedExperiment) will have range data dropped, the response about warning about unsupported classes is not a complete discussion.

5. Given these issues, before considering the manuscript again I would want to see a) MuData and MuDataSeurat *accepted* into Bioconductor and CRAN, and b) a series of actual cross-language examples implemented, where real-world datasets are imported and proof-of-concept basic analysis is performed in Python, R, and Julia. Figure 3 is a demonstration of some generic analyses, but based on the vignette links provided in the Methods, I believe they are only performed in the Python implementation muon, with not guidance on importing these data into R or Julia. I think the promise of cross-language support is a major selling point of Muon/Mudata, and I want it to succeed at this, but I am currently doubtful that using it to share multimodal data between Python, R, and Julia on real-world data would be successful in practice. Session Info: > devtools::session_info() ─ Session info ────────────────────────────────────────────────────────────────────────────────────────────────────────────────────────────── hash: volleyball, flag: United Nations, man astronaut: light skin tone setting value version R version 4.1.2 os Ubuntu 18.04.6 LTS system x86_64, linux-gnu ui RStudio language (EN) pandoc 2.11.4 @ /usr/lib/rstudio-server/bin/pandoc/ (via rmarkdown) ─ Packages ─────────────────────────────────────────────────────────────────────────────────────────────────────────────────────────────────── package * version date (UTC) lib source abind 1.4-5 2016-07-21 [1] CRAN (R 4.1.0) AnnotationDbi 1.56.2 2021-11-09 [1] Bioconductor AnnotationHub 3.2.0 2021-10-26 [1] Bioconductor assertthat 0.2.1 2019-03-21 [1] CRAN (R 4.1.0) Biobase * 2.54.0 2021-10-26 [1] Bioconductor BiocFileCache 2.2.0 2021-10-26 [1] Bioconductor BiocGenerics * 0.40.0 2021-10-26 [1] Bioconductor BiocManager 1.30.16 2021-06-15 [1] CRAN (R 4.1.2) BiocVersion 3.14.0 2021-05-19 [1] Bioconductor Biostrings 2.62.0 2021-10-26 [1] Bioconductor bit 4.0.4 2020-08-04 [1] CRAN (R 4.1.0) bit64 4.0.5 2020-08-30 [1] CRAN (R 4.1.0) bitops 1.0-7 2021-04-24 [1] CRAN (R 4.1.0) blob 1.2.2 2021-07-23 [1] CRAN (R 4.1.0) cachem 1.0.6 2021-08-19 [1] CRAN (R 4.1.1) callr 3.7.0 2021-04-20 [1] CRAN (R 4.1.0) cli 3.1.0 2021-10-27 [1] CRAN (R 4.1.1) cluster 2.1.2 2021-04-17 [2] CRAN (R 4.0.5) codetools 0.2-18 2020-11-04 [2] CRAN (R 4.0.3) colorspace 2.0-2 2021-06-24 [1] CRAN (R 4.1.0) cowplot 1.1.1 2020-12-30 [1] CRAN (R 4.1.0) crancache 0.0.0.9001 2020-06-26 [1] Github (r-lib/crancache@7ea4e47) cranlike 1.0.2 2018-11-26 [1] CRAN (R 4.1.0) crayon 1.4.2 2021-10-29 [1] CRAN (R 4.1.2) curatedTCGAData * 1.16.0 2021-10-30 [1] Bioconductor curl 4.3.2 2021-06-23 [1] CRAN (R 4.1.0) data.table 1.14.2 2021-09-27 [1] CRAN (R 4.1.1) DBI 1.1.1 2021-01-15 [1] CRAN (R 4.1.0) dbplyr 2.1.1 2021-04-06 [1] CRAN (R 4.1.0) debugme 1.1.0 2017-10-22 [1] CRAN (R 4.1.0) DelayedArray 0.20.0 2021-10-26 [1] Bioconductor deldir 1.0-6 2021-10-23 [1] CRAN (R 4.1.1) desc 1.4.0 2021-09-28 [1] CRAN (R 4.1.1) devtools 2.4.2 2021-06-07 [1] CRAN (R 4.1.0) digest 0.6.28 2021-09-23 [1] CRAN (R 4.1.1) dplyr 1.0.7 2021-06-18 [1] CRAN (R 4.1.0) ellipsis 0.3.2 2021-04-29 [1] CRAN (R 4.1.0) evaluate 0.14 2019-05-28 [1] CRAN (R 4.1.0) ExperimentHub 2.2.0 2021-10-26 [1] Bioconductor fansi 0.5.0 2021-05-25 [1] CRAN (R 4.1.0) fastmap 1.1.0 2021-01-25 [1] CRAN (R 4.1.0) filelock 1.0.2 2018-10-05 [1] CRAN (R 4.1.0) fitdistrplus 1.1-6 2021-09-28 [1] CRAN (R 4.1.1) fs 1.5.0 2020-07-31 [1] CRAN (R 4.1.0) future 1.23.0 2021-10-31 [1] CRAN (R 4.1.2) future.apply 1.8.1 2021-08-10 [1] CRAN (R 4.1.0) generics 0.1.1 2021-10-25 [1] CRAN (R 4.1.1) GenomeInfoDb * 1.30.0 2021-10-26 [1] Bioconductor GenomeInfoDbData 1.2.7 2021-10-28 [1] Bioconductor GenomicRanges * 1.46.1 2021-11-18 [1] Bioconductor ggplot2 3.3.5 2021-06-25 [1] CRAN (R 4.1.0) ggrepel 0.9.1 2021-01-15 [1] CRAN (R 4.1.0) ggridges 0.5.3 2021-01-08 [1] CRAN (R 4.1.0) globals 0.14.0 2020-11-22 [1] CRAN (R 4.1.0) glue 1.5.0 2021-11-07 [1] CRAN (R 4.1.2) goftest 1.2-3 2021-10-07 [1] CRAN (R 4.1.1) gridExtra 2.3 2017-09-09 [1] CRAN (R 4.1.0) gtable 0.3.0 2019-03-25 [1] CRAN (R 4.1.0) HDF5Array 1.22.1 2021-11-14 [1] Bioconductor hdf5r 1.3.5 2021-11-15 [1] CRAN (R 4.1.2) htmltools 0.5.2 2021-08-25 [1] CRAN (R 4.1.1) htmlwidgets 1.5.4 2021-09-08 [1] CRAN (R 4.1.1) httpuv 1.6.3 2021-09-09 [1] CRAN (R 4.1.1) httr 1.4.2 2020-07-20 [1] CRAN (R 4.1.0) ica 1.0-2 2018-05-24 [1] CRAN (R 4.1.0) igraph 1.2.9 2021-11-23 [1] CRAN (R 4.1.2) interactiveDisplayBase 1.32.0 2021-10-26 [1] Bioconductor IRanges * 2.28.0 2021-10-26 [1] Bioconductor irlba 2.3.3 2019-02-05 [1] CRAN (R 4.1.0) jsonlite 1.7.2 2020-12-09 [1] CRAN (R 4.1.0) KEGGREST 1.34.0 2021-10-26 [1] Bioconductor KernSmooth 2.23-20 2021-05-03 [2] CRAN (R 4.0.5) knitr 1.36 2021-09-29 [1] CRAN (R 4.1.1) later 1.3.0 2021-08-18 [1] CRAN (R 4.1.1) lattice 0.20-45 2021-09-22 [2] CRAN (R 4.1.1) lazyeval 0.2.2 2019-03-15 [1] CRAN (R 4.1.0) leiden 0.3.9 2021-07-27 [1] CRAN (R 4.1.0) lifecycle 1.0.1 2021-09-24 [1] CRAN (R 4.1.1) listenv 0.8.0 2019-12-05 [1] CRAN (R 4.1.0) lmtest 0.9-39 2021-11-07 [1] CRAN (R 4.1.2) magrittr 2.0.1 2020-11-17 [1] CRAN (R 4.1.0) MASS 7.3-54 2021-05-03 [2] CRAN (R 4.0.5) Matrix * 1.3-4 2021-06-01 [2] CRAN (R 4.1.0) MatrixGenerics * 1.6.0 2021-10-26 [1] Bioconductor matrixStats * 0.61.0 2021-09-17 [1] CRAN (R 4.1.1) memoise 2.0.0 2021-01-26 [1] CRAN (R 4.1.0) mgcv 1.8-38 2021-10-06 [2] CRAN (R 4.1.1) mime 0.12 2021-09-28 [1] CRAN (R 4.1.1) miniUI 0.1.1.1 2018-05-18 [1] CRAN (R 4.1.0) MuData * 0.99.0 2021-11-26 [1] Github (PMBio/MuDataMAE@bc1ea75) MuDataSeurat * 0.0.0.9000 2021-11-26 [1] Github (PMBio/MuDataSeurat@c4b54d1) MultiAssayExperiment * 1.20.0 2021-10-26 [1] Bioconductor munsell 0.5.0 2018-06-12 [1] CRAN (R 4.1.0) nlme 3.1-153 2021-09-07 [1] CRAN (R 4.1.1) parallelly 1.29.0 2021-11-21 [1] CRAN (R 4.1.2) parsedate 1.2.1 2021-04-20 [1] CRAN (R 4.1.0) patchwork 1.1.1 2020-12-17 [1] CRAN (R 4.1.0) pbapply 1.5-0 2021-09-16 [1] CRAN (R 4.1.1) pillar 1.6.4 2021-10-18 [1] CRAN (R 4.1.1) pkgbuild 1.2.0 2020-12-15 [1] CRAN (R 4.1.0) pkgconfig 2.0.3 2019-09-22 [1] CRAN (R 4.1.0) pkgload 1.2.3 2021-10-13 [1] CRAN (R 4.1.1) plotly 4.10.0 2021-10-09 [1] CRAN (R 4.1.1) plyr 1.8.6 2020-03-03 [1] CRAN (R 4.1.1) png 0.1-7 2013-12-03 [1] CRAN (R 4.1.0) polyclip 1.10-0 2019-03-14 [1] CRAN (R 4.1.0) prettyunits 1.1.1 2020-01-24 [1] CRAN (R 4.1.0) processx 3.5.2 2021-04-30 [1] CRAN (R 4.1.0) promises 1.2.0.1 2021-02-11 [1] CRAN (R 4.1.0) ps 1.6.0 2021-02-28 [1] CRAN (R 4.1.0) purrr 0.3.4 2020-04-17 [1] CRAN (R 4.1.0) R6 2.5.1 2021-08-19 [1] CRAN (R 4.1.1) RaggedExperiment * 1.18.0 2021-10-26 [1] Bioconductor RANN 2.6.1 2019-01-08 [1] CRAN (R 4.1.0) rappdirs 0.3.3 2021-01-31 [1] CRAN (R 4.1.0) RColorBrewer 1.1-2 2014-12-07 [1] CRAN (R 4.1.0) Rcpp 1.0.7 2021-07-07 [1] CRAN (R 4.1.0) RcppAnnoy 0.0.19 2021-07-30 [1] CRAN (R 4.1.0) RCurl 1.98-1.5 2021-09-17 [1] CRAN (R 4.1.1) rematch2 2.1.2 2020-05-01 [1] CRAN (R 4.1.0) remotes 2.4.1 2021-09-29 [1] CRAN (R 4.1.1) reshape2 1.4.4 2020-04-09 [1] CRAN (R 4.1.0) reticulate 1.22 2021-09-17 [1] CRAN (R 4.1.1) rhdf5 * 2.38.0 2021-10-26 [1] Bioconductor rhdf5filters 1.6.0 2021-10-26 [1] Bioconductor Rhdf5lib 1.16.0 2021-10-26 [1] Bioconductor rlang 0.4.12 2021-10-18 [1] CRAN (R 4.1.1) rmarkdown 2.11 2021-09-14 [1] CRAN (R 4.1.1) ROCR 1.0-11 2020-05-02 [1] CRAN (R 4.1.0) rpart 4.1-15 2019-04-12 [2] CRAN (R 4.0.0) rprojroot 2.0.2 2020-11-15 [1] CRAN (R 4.1.0) RSQLite 2.2.8 2021-08-21 [1] CRAN (R 4.1.1) rstudioapi 0.13 2020-11-12 [1] CRAN (R 4.1.0) Rtsne 0.15 2018-11-10 [1] CRAN (R 4.1.0) S4Vectors * 0.32.3 2021-11-21 [1] Bioconductor scales 1.1.1 2020-05-11 [1] CRAN (R 4.1.0) scattermore 0.7 2020-11-24 [1] CRAN (R 4.1.0) sctransform 0.3.2 2020-12-16 [1] CRAN (R 4.1.0) sessioninfo 1.2.1 2021-11-02 [1] CRAN (R 4.1.2) Seurat 4.0.5 2021-10-17 [1] CRAN (R 4.1.1) SeuratObject 4.0.4 2021-11-23 [1] CRAN (R 4.1.2) shiny 1.7.1 2021-10-02 [1] CRAN (R 4.1.1) SingleCellExperiment 1.16.0 2021-10-26 [1] Bioconductor spatstat.core 2.3-1 2021-11-02 [1] CRAN (R 4.1.2) spatstat.data 2.1-0 2021-03-21 [1] CRAN (R 4.1.0) spatstat.geom 2.3-0 2021-10-09 [1] CRAN (R 4.1.1) spatstat.sparse 2.0-0 2021-03-16 [1] CRAN (R 4.1.0) spatstat.utils 2.2-0 2021-06-14 [1] CRAN (R 4.1.0) stringi 1.7.5 2021-10-04 [1] CRAN (R 4.1.1) stringr 1.4.0 2019-02-10 [1] CRAN (R 4.1.1) SummarizedExperiment * 1.24.0 2021-10-26 [1] Bioconductor survival 3.2-13 2021-08-24 [2] CRAN (R 4.1.1) tensor 1.5 2012-05-05 [1] CRAN (R 4.1.0) testthat 3.1.0 2021-10-04 [1] CRAN (R 4.1.1) tibble 3.1.6 2021-11-07 [1] CRAN (R 4.1.2) tidyr 1.1.4 2021-09-27 [1] CRAN (R 4.1.1) tidyselect 1.1.1 2021-04-30 [1] CRAN (R 4.1.0) usethis 2.1.3 2021-10-27 [1] CRAN (R 4.1.1) utf8 1.2.2 2021-07-24 [1] CRAN (R 4.1.0) uwot 0.1.10 2020-12-15 [1] CRAN (R 4.1.0) vctrs 0.3.8 2021-04-29 [1] CRAN (R 4.1.0) viridisLite 0.4.0 2021-04-13 [1] CRAN (R 4.1.0) withr 2.4.2 2021-04-18 [1] CRAN (R 4.1.0) xfun 0.28 2021-11-04 [1] CRAN (R 4.1.2) xtable 1.8-4 2019-04-21 [1] CRAN (R 4.1.0) XVector 0.34.0 2021-10-26 [1] Bioconductor yaml 2.2.1 2020-02-01 [1] CRAN (R 4.1.0) zlibbioc 1.40.0 2021-10-26 [1] Bioconductor zoo 1.8-9 2021-03-09 [1] CRAN (R 4.1.0)

**Authors Response**

**Point-by-point responses to the reviewers’ comments:**

Some improvements have been made, including separating MuData and MuDataSeurat, and maintaining the sampleMap when writing a MultiAssayExperiment. But I remain concerned about usability of the R software, and the commitment to submitting it to Bioconductor and CRAN, and to providing the level of documentation and ongoing development that would be required to build and maintain a user base. The pull request apparently referenced in the response (https://github.com/grimbough/rhdf5/pull/95) was merged into rhdf5 on Sept 7, but in the couple of months since, MuData has still not been submitted to Bioconductor and MuDataSeurat still has not been submitted to CRAN. With no Continuous Integration in use, I had to download and check the packages locally to determine that these packages still contain a number of issues making them difficult to use or assess. Using R 4.1.2 and Bioconductor 3.14 (full devtools::session_info() at the end).

*Thank you for your attention to the details regarding the R packages functionality!*

*Regarding the pull requests that we submitted to rhdf5 and the progress on them, we were referring to https://github.com/grimbough/rhdf5/pull/96 as well, which has also been recently merged to the developmental branch of rhdf5. We now have initiated the submission process to Bioconductor (*[*https://github.com/Bioconductor/Contributions/issues/2453*](https://github.com/Bioconductor/Contributions/issues/2453)*).*

*We have also set up continuous integration for all 3 major platforms (macOS, Linux, Windows) to build and test both R packages as well to automatically build their vignettes and documentation. We believe that addresses most of the points, which we also reply to in detail below.*

1. Checking MuData

\# for MuData:

git clone https://github.com/pmbio/MuDataMAE

cd MuDataMAE

R CMD check .

* checking for file ‘./DESCRIPTION’ ... ERROR

Required fields missing or empty:

‘Author’ ‘Maintainer’

*The ‘Author’ and ‘Maintainer’ fields are generated by ‘R CMD build’ from the ‘Authors@R’ field. So as far as this message is concerned, the proper workflow is to run ‘R CMD build’ first and then to run R CMD check on the generated tarball. This is also stated by the official “Writing R extensions” manual: “It is strongly recommended that the final checks are run on a tar archive prepared by R CMD build.“*

*To automate checking the R package we have implemented ‘R CMD check’ as a workflow with GitHub actions so one can check the package is expected to work on their platform:*

[*https://github.com/PMBio/MuDataMAE/blob/master/.github/workflows/R-CMD-check.yaml*](https://github.com/PMBio/MuDataMAE/blob/master/.github/workflows/R-CMD-check.yaml)*.*

$ R CMD BUILD . --no-build-vignettes ## note erroneously capitalized BUILD in response to

reviewers

/usr/lib/R/bin/Rcmd: 64: exec: BUILD: not found

*Here we would like to note that some platforms permit case-insensitive syntax so that both BUILD and build (as well as Build, etc.) can be used.*

$ R CMD build . --no-build-vignettes ##corrected BUILD -> build

* checking for file ‘./DESCRIPTION’ ... OK

* preparing ‘MuData’:

* checking DESCRIPTION meta-information ... OK

* checking for LF line-endings in source and make files and shell scripts

* checking for empty or unneeded directories

Omitted ‘LazyData’ from DESCRIPTION

* building ‘MuData_0.99.0.tar.gz’

If R CMD build is run with vignettes, the vignette does not build, ending with the following error, meaning the code can’t just be run by a user:

E> Quitting from lines 116-118 (Getting-Started.Rmd)

E> Error: processing vignette 'Getting-Started.Rmd' failed with diagnostics:

E> failed to open HDF5 file '/tmp/RtmpDZRrLM/Rbuild54226f0af12c/MuData/vignettes/miniacc.h5mu'

E> --- failed re-building ‘Getting-Started.Rmd’

*An issue with these lines of the vignette is fixed.*

*As part of the documentation, the R vignettes are now also automatically compiled using continuous integration with GitHub Actions and are available at https://ilia-kats.github.io/MuData/.*

There are some significant BiocCheck notes (leaving out the less significant notes):

[1]	"'LazyData:' in the 'DESCRIPTION' should be set to false or removed"

[2]	"Use accessors; don't access S4 class slots via '@' in examples/vignettes."

[3]	"Avoid '<<-' if possible (found 1 times)"

[4]	"Avoid 'suppressWarnings'/'*Messages' if possible (found 1 times)"

*While notes seem to provide additional information to developers, we have addressed them in the latest MuData R package version. In particular, we fixed points [1] – [3] from this list. [4] is apparently new in BioConductor 3.14 (Oct 27). Removing the suppressWarnings call will emit numerous warnings when reading files in backed mode, because HDF5Array assumes that it has exclusive access to the file. While in principle, it would be possible to re-define suppressWarnings() to silence BiocCheck here, this does not seem necessary as the BiocCheck vignette states:*

*NOTE: Not necessarily something bad, just something we wanted to point out. package authors don’t need to take action on these, but they can.*

2. MuDataSeurat

The MuDataSeurat vignette uses two packages (SeuratData, BiocStyle) that are undeclared in the DESCRIPTION Suggests field, causing errors on a fresh containerized check. SeuratData is not in CRAN, so it requires defining remotes or googling to find where to install the package from. After resolving this, R CMD check on MuDataSeurat still has 6 warnings and 6 notes that would have to be resolved for it to be accepted into CRAN. There is an unusual “Warning in file.copy(pkgdir, vd2, recursive = TRUE) : too deep nesting”, along with more routine ones about undocumented code objects and calls to unimported functions not defined in the global environment.

*We added BiocStyle and SeuratData as suggested libraries to the DESCRIPTION file, which means users can now install these packages easier in case they might want to compile the vignettes. We note that these packages are required for building vignettes only, and SeuratData is only used as a suitable library from the Seurat ecosystem to obtain data in the form of Seurat objects and is not a strict dependency.*

*Building and checking the package is now set up with continuous integration, and R CMD check output can be checked across all major OS platforms: https://github.com/PMBio/MuDataSeurat/actions/workflows/R-CMD-check.yaml.*

3. coad example

The coad example from my previous review now gives some informative warnings about skipping RaggedExperiment objects, but it still gives a cryptic error in the end “Error in assign(mname, def, where)”:

> WriteH5MU(coad, "coad_test.mu")

Error in assign(mname, def, where) :

cannot add bindings to a locked environment

In addition: Warning messages:

1:	In .local(object, file, overwrite, ...) :

Objects of class RaggedExperiment are currently unsupported, skipping...

2:	In .local(object, file, overwrite, ...) :

Objects of class RaggedExperiment are currently unsupported, skipping...

3:	In .local(object, file, overwrite, ...) :

Objects of class RaggedExperiment are currently unsupported, skipping...

4:	In .local(object, file, overwrite, ...) :

Objects of class RaggedExperiment are currently unsupported, skipping...

This error also occurs with the object after removing the RaggedExperiment objects, suggested the authors didn’t check whether the example would now run after dropping RaggedExperiment objects.

*Thank you, we were able to reproduce this error (of note, this only occurred when not loading the package via devtools::load_all() and hence was missed in the previous iteration). This problem should be fixed now in the latest version of MuData. We have also added test cases based on this example, which we run as part of the continuous integration set up for the MuData R package.*

4. discussion about genomic coordinate-based data

“The software does not seem to support genomic coordinate-based data, which should be discussed and acknowledged as a shortcoming.” – this is now discussed in the response to reviewers, but still not in the manuscript. As a note, supported data classes (e.g. RangedSummarizedExperiment) will have range data dropped, the response about warning about unsupported classes is not a complete discussion.

*We have now added another warning about range data being potentially dropped when working with RangedSummarizedExperiment.*

*As both the framework and the manuscript are focused on the multimodal nature of the data addressing the issues around its management and analysis, we chose not to discuss individual modalities and their specifics. We would be glad to see improved support for coordinate-based data coming to more ecosystems, and it seems that this discussion is more geared towards AnnData and new potential AnnData-like formats and/or is related to external Python libraries such as pyranges rather than MuData itself. In AnnData, RaggedExperiment features are already being implemented (pull/647). Moreover, coordinate-based data represented as intervals (such as peaks or bins in ATAC-seq data) can already be operated in the AnnData format and with muon as exemplified in muon tutorials. Having said this, we have included the following sentence in the Discussions section, highlighting more explicit support for genomic-coordinate based data is an area of future developments:*

*<...> Other examples include explicit support for genomic-coordinate based assays [Stovner et al., 2020] or assays with spatial coordinates [Palla et al., 2021].*

5. Given these issues, before considering the manuscript again I would want to see a) MuData and MuDataSeurat *accepted* into Bioconductor and CRAN, and b) a series of actual cross-language examples implemented, where real-world datasets are imported and proof-of-concept basic analysis is performed in Python, R, and Julia. Figure 3 is a demonstration of some generic analyses, but based on the vignette links provided in the Methods, I believe they are only performed in the Python implementation muon, with not guidance on importing these data into R or Julia. I think the promise of cross-language support is a major selling point of Muon/Mudata, and I want it to succeed at this, but I am currently doubtful that using it to share multimodal data between Python, R, and Julia on real-world data would be successful in practice.

*To address (a), we have prepared the MuData R package for Bioconductor submission by ensuring BiocCheck() passes without errors, warnings, or significant notes. It is tested against bioc-devel so that it is compatible with the next Bioconductor release (Spring 2022), and we have submitted MuData to Bioconductor.*

*Regarding (b), since the cross-language functionality revolves around H5MU and H5AD files, existing vignettes already provide a demonstration of how these files can be imported into R (e.g. to form MultiAssayExperiment objects or Seurat objects) or Julia (including examples in readme or doc pages).*

*To improve upon it, we have added vignettes that demonstrate how multimodal datasets from H5MU files can be loaded, operated and written in R. To address the continuity with analyses performed in Python, in the new MuDataSeurat vignette we use the output of the CITE-seq data analysis with muon (for practical reasons, we use a subset of cells and features):*

*https://pmbio.github.io/MuDataSeurat/articles/PBMC-CITE-seq.html.*

*In the new MuData vignette for Bioconductor we showcase a complementary scenario with data being imported with the SingleCellMultiModal or the CiteFuse Bioconductor package, processed utilising the scater package and saved as H5MU file, allowing further analysis performed with other tools:*

*https://ilia-kats.github.io/MuData/articles/Blood-CITE-seq.html,*

*https://ilia-kats.github.io/MuData/articles/Cord-Blood-CITE-seq.html.*

*We hope these examples help to sufficiently illustrate the cross-language benefit of MuData.*
